# Efficacy of Bendamustine, Pomalidomide, and Dexamethasone (BPD) Regimen in Relapsed/Refractory Extramedullary Myeloma: A Retrospective Single-Centre Study, Real-Life Experience

**DOI:** 10.3390/hematolrep15030048

**Published:** 2023-08-02

**Authors:** İbrahim Halil Açar, Birol Güvenç

**Affiliations:** 1 Department of Hematology, Osmaniye State Hospital, 80000 Osmaniye, Turkey; 2 Department of Hematology, Çukurova University, 01330 Adana, Turkey; guvenc.birol@gmail.com

**Keywords:** relapsed/refractory extramedullary myeloma, bendamustine, pomalidomide, dexamethasone, treatment outcomes, multiple myeloma

## Abstract

Background and Objectives: Relapsed/refractory extramedullary myeloma (RREMM) is an uncommon and aggressive subtype of multiple myeloma defined by plasma cell proliferation outside the bone marrow. Therapeutic options for RREMM are limited, and the prognosis is generally unfavorable. This research aimed to assess the effectiveness of the bendamustine, pomalidomide, and dexamethasone (BPD) regimen in patients with RREMM. Material and Methods: We carried out a retrospective investigation of 11 RREMM patients who underwent BPD treatment. The primary endpoint was progression-free survival. The secondary endpoints of the study were two-year survival and overall response rate (ORR). We analyzed the sociodemographic and clinical features of the patients. Results: The average age of the patients was 62 years. They had a median of four prior treatment lines, and eight patients had previously received autologous stem-cell transplantation. After eight BPD treatment cycles, the ORR stood at 54%, with one very good partial response (VGPR), five partial responses (PR), three progressive diseases (PD), and two stable diseases (SD). The median follow-up was 15 months, with a two-year PFS rate of 71.3% and a two-year survival rate of 81.8%. Conclusions: The BPD regimen demonstrated promising effectiveness in RREMM patients, yielding favorable ORR and survival rates. To corroborate these findings and explore additional treatment alternatives for this patient group, larger prospective studies are required.

## 1. Introduction

Multiple myeloma (MM) is a hematological malignancy that arises from plasma cells, which play a crucial role in antibody production and immune responses [1]. The median age at diagnosis is approximately 69 years, and the incidence rate stands at around 6.3 cases per 100,000 people per year in the United States (US) [2,3]. MM constitutes about 1% of all cancers and 10% of hematological cancers, with an estimated 34,920 new cases and 12,410 deaths in 2021 in the US [4,5].

Relapsed/refractory extramedullary myeloma (RREMM) is an uncommon and aggressive subtype of MM characterized by malignant plasma cell proliferation outside the bone marrow, leading to tumor formation in various organs and tissues [6]. Primary extramedullary myeloma is present at diagnosis, while secondary extramedullary myeloma emerges during disease progression or relapse [7]. The incidence of extramedullary myeloma at diagnosis ranges between 7 and 18%, rising to 30–50% throughout the disease course [8]. Risk factors for RREMM development include a high tumor burden, high-risk cytogenetics (e.g., del(17p), t(4;14), or t(14;16)), a previous history of MM, and disease progression or relapse following autologous stem cell transplantation [9]. The prognosis is generally unfavorable, with a median overall survival of around 8–12 months [10].

Bendamustine is a distinctive bifunctional alkylating agent that merges the mechanisms of action of both alkylating agents and purine analogs [11]. In real-life experience in relapsed/refractory extramedullary myeloma (RRMM) patients, bendamustine has shown impressive and promising results [12]. In this study, three-drug regimens containing bendamustine, steroids, and novel agents showed superior properties and a safe toxicity profile. Bendamustine has been tested in RREMM patients, and real-life data on its efficacy are scarce. We hypothesized that a bendamustine, pomalidomide, and dexamethasone treatment might be an effective treatment option for patients who had previously received bortezomib and lenalidomide and were lenalidomide-resistant, and had received at least two lines of therapy. This study aimed to evaluate the role of a bendamustine, pomalidomide and dexamethasone (BPD) regimen in patients with RREMM as a real-life experience.

## 2. Material and Methods

The study was retrospective and single-armed, and informed consent was obtained from all patients (*n* = 11) before starting the regimen. Between 2019 and 2022, 129 multiple-myeloma patients were screened. Among these patients, 11 RREMM patients (all patients had EMM at the time of diagnosis) who received 2 or more treatments including bortezomib and lenalidomide and met the criteria of having an ECOG score of <2 were included in the study. These 11 RREMM patients were a subset of all RRMM patients treated at the Hematology Clinic of Çukurova University Faculty of Medicine from 2019 to 2022. After several treatment lines, all patients had the extramedullary disease. The age of the patients ranged from 48 to 70 years and there was no liver or kidney dysfunction in the pre-treatment evaluation.

Patient’s age, gender, ECOG performance score, number of previous treatment lines, R-ISS stage at diagnosis, extramedullary involvement sites, autologous stem cell transplantation information, response to treatment, and survival information were recorded in case report forms.

EMM was defined as the accumulation of clonal plasma cells in regions other than the bone marrow in a patient with MM. To avoid confusion in categorizing EMD, the EMD classification introduced by Bansal et al. was used (Table 1).

**Table 1:** EMD classification. Reprinted with permission from Ref. [13]. 2021, Bansal R.

Type of EMD	Definition
Solitary plasmacytoma (SP) with no marrow involvement	Biopsy-proven bone or soft tissue lesion with evidence of clonal plasma cells. However, marrow has no clonal PCs and no additional abnormality on imaging and absence of CRAB criteria.
Solitary plasmacytoma with minimal marrow involvement	SP with <10% clonal BMPC.
Bone-associated EMD with MM (EMM)	Soft tissue mass arising from bone lesions and growing contiguously.
Bone-independent EMD with MM (EMM)	Isolated extra-osseous plasma cell tumors not contiguous with bone lesions.
Organ-infiltrating EMD	CNS myeloma, diffuse liver involvement etc.

**Figure 1:**
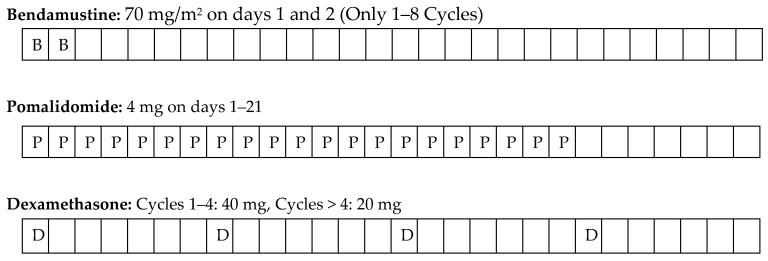
Treatment protocol.

The BPD regimen was Bendamustine for 28 days each cycle, intravenous (IV) at a dose of 70 mg/m^2^ on days 1 and 2 of the cycle, Pomalidomide 4 mg orally once a day on days 1 to 21, and dexamethasone 40 mg on days 1, 8, 15 and 22, which was taken orally once a day. After four cycles, dexamethasone treatment was reduced to 20 mg. To reduce the risk of bendamustine-related toxicity and secondary malignancy, bendamustine was discontinued in patients receiving 8 cycles and continued with pomalidomide-dexamethasone (PD) treatment alone (Figure 1).

The BPD regimen was administered until unacceptable toxicity or progression developed. Progression-free survival (PFS) was defined as the time from the start of BPD treatment to disease progression or death from any cause. The primary endpoint was PFS. In addition, the two-year survival rate was calculated to determine the percentage of people who were alive two years after the initiation of BPD treatment in RREMM patients. The secondary endpoints of the study were two-year survival rate and overall response rate (ORR). Response to treatment was evaluated with biochemical analyses, bone marrow biopsy, and PET-CT after every 4 cycles. International Myeloma Working Group consensus 2016 criteria were used to assess response to treatment [14].

The study protocol was approved by the Ethics Committee of Çukurova University Faculty of Medicine (Decision No. 41, dated 4 February 2023) and adhered to the Declaration of Helsinki and Good Clinical Practice guidelines issued by the International Conference on Harmonization.

## 3. Statistical Analysis

Statistical analyses were carried out using “IBM SPSS Statistics for Windows. Version 25.0 (Statistical Pac-kage for the Social Sciences, IBM Corp., Armonk, NY, USA)”. Descriptive statistics are presented as *n* and % for categorical variables and mean ± SD or median (min-max) for continuous variables. Finally, Kaplan–Meier method was used to give progression free survival (PFS) times.

## 4. Results

Sociodemographic and clinical characteristics of the patients are given in Table 1. The mean age of the patients was 62 years. A total of 11 patients who received BPD treatment were included in the study. Six of the patients were female and five were male. Patients with an ECOG score of one comprised 54% of the cases. When the patients were examined according to the R-ISS stage at the time of diagnosis, nine of the cases had stage 3 and two had stage 2 disease at the time of diagnosis. At the time of diagnosis, two patients had Del 17p mutation and one patient had t(4;14). All patients had previously received bortezomib and lenalidomide treatment. Eight of the patients had previously undergone autologous bone marrow transplant (ASCT), and patients with BPD treatment had received a median of four rows of treatment for myeloma (Table 2). There was no difference in responses to BPD whether patients had previously received ASCT or not. The mean/median time between prior step and initiation of BPD treatment were 3.6/3.1 months, respectively. No serious toxicity was observed during the administration of BPD, necessitating discontinuation of the treatment. The most common side effects during BPD treatment were neutropenia (*n* = 4) and anemia (*n* = 2), which were manageable (grade 1 and grade 2). Of the 11 patients included in the study, 10 received eight cycles of BPD therapy. Only one patient had death due to bone marrow failure associated with disease progression after the fourth cycle. In the evaluation of response after eight cycles of BPD treatment, a response consistent with VGPR in one patient, PR in five patients, PD in three patients, and SD in two patients was observed. The overall response rate (ORR) was 54%. At the end of follow-up, the two-year survival rate was 81.8%.

**Figure 2:**
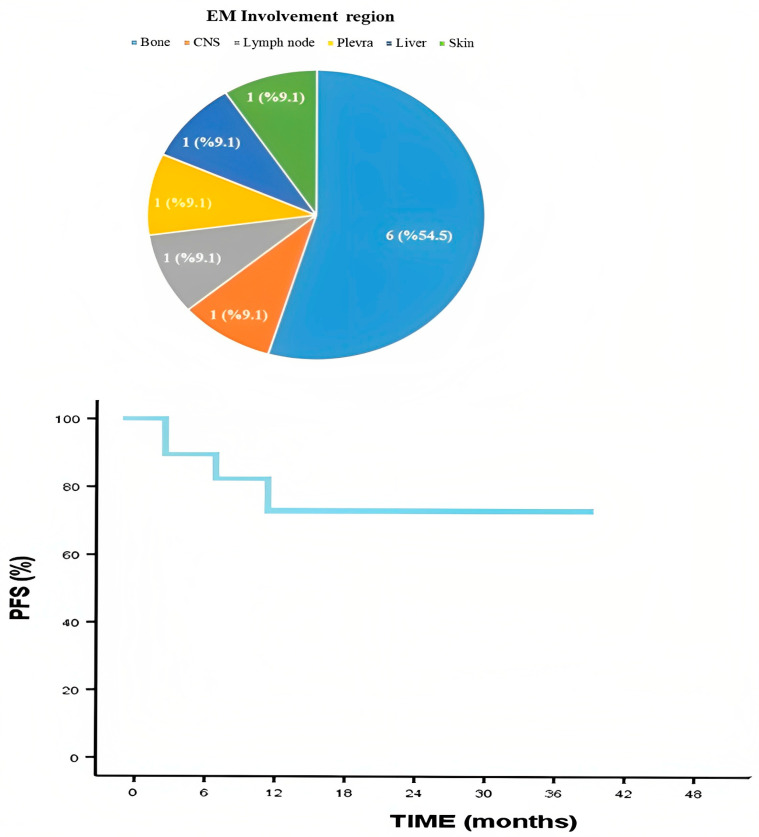
Extramedullary involvement region and progression-free survival.

When the patients were examined according to the EMM involvement regions, six patients had cortical bone, one patient had CNS, one patient had skin, one patient had lymph node, one patient had liver, and one patient had pleural involvement. PFS, which is an indicator of the power of the treatment to keep the disease under control, was also calculated. As seen in Figure 2, the median follow-up period was 15 months, and the two-year progression-free survival rate was 71.3%.

## 5. Discussion

RREMM is a myeloma entity that with a very poor prognosis. The presence of EMM with high-risk cytogenetic features and avoidance of apoptosis make the disease aggressive and thus its response to treatment is greatly reduced. Plasma cells proliferate more rapidly in EMD and there is resistance to standard cytotoxic treatments. Various combination therapies have been tried to reduce the devastating effects of the disease. The available evidence in the treatment of EMM is generally from retrospective studies. To contextualize our results, we will juxtapose them with findings from other research in the literature that have assessed various treatment strategies in RREMM and RRMM patients. Clinical trials in RREMM patients are often shaped by the results obtained in RRMM patients. Our investigation builds upon their work by examining a novel treatment for RREMM, which may provide improved outcomes for this specific patient population.

Kumar and colleagues conducted a phase II trial, which combined bendamustine, pomalidomide, and dexamethasone for the treatment of RRMM. In this phase 2 study investigating the efficacy of BPD treatment in RRMM patients, BPD showed better results in EMM patients. It has been reported that patients with EMM involvement show more efficacy than those without EMM involvement. Therefore, the interest in bendamustine combinations has increased in EMM cases. Toxicity in this study was manageable and generally consisted of haematological adverse events such as neutropenia and anemia. Their findings demonstrated encouraging results regarding the ORR and PFS [15]. The effectiveness of our investigational treatment was comparable, suggesting that our regimen might be a viable option for RREMM patients.

Musto and colleagues carried out research on the use of bendamustine in RRMM, highlighting its potential role for patients who had exhausted other treatment options [16]. In this study, it was emphasized that combination treatments containing bendamustine had a synergistic effect by overcoming non-cross-resistance in RRMM patients. The most common side effect was also hematological toxicities, and grade 3–4 hematological toxicities occurred in 44 (56%) patients. It was also stated that the bendamustine-based combination therapies used had an acceptable toxicity profile.

Richardson et al. conducted a randomized phase 2 study comparing pomalidomide alone or in combination with low-dose dexamethasone in RRMM patients [17]. Grade 3–4 neutropenia was the most common side effect. The combination of pomalidomide with low-dose dexamethasone significantly improved PFS in patients who had previously received multiple prior therapies compared to pomalidomide alone and has been suggested as a new treatment option.

Rodon et al. reported a phase II study of bendamustine, bortezomib, and dexamethasone (BVD) as a second-line treatment for elderly patients with MM [18]. The most common adverse events are non-hematological toxicities. BVD treatment has been shown to be a good candidate in elderly patients, especially during the first relapse. It has been recommended as an effective and safe combination therapy due to its rapid and high response (approximately 70%). The study extends these findings to RREMM patients, providing an additional treatment option for this challenging population.

Ludwig et al. reported that in another phase II study, BVD was administered to 79 patients with RRMM [19]. Common grade 3–4 side effects were thrombocytopenia and infections. The median follow-up was 13.7 months, with an ORR of 60.8%. In this study, BVD was shown to be active and well tolerated in patients with RRMM.

The efficacy and safety of the bendamustine, lenalidomide, and dexamethasone (BLD) combination was tested in a phase 2 study by Lentzsch et al. with 29 patients. The most common side effects were also hematological toxicities such as neutropenia, thrombocytopenia and anemia. The BLD combination was feasible and highly active in RRMM. This combination has been proposed as an effective and safe option in patients with RRMM [20].

When the studies were compared, the patients in the study of Kumar et al., had a lower median age (Table 3). When the median (median age 66 years (range 48–70 years)) age of our patient population is compared with the table above, it is similar to RRMM studies. The median follow-up period varies between 8 and 15.7 months in the studies mentioned (Table 3), and the median follow-up period of our study is relatively long. Results from these studies showed that the prognosis is better in patients receiving a triple-combination therapy.

The research for the optimal treatment in the management of RREMM continues. In RRMM, the number of lines of treatment that patients have received before is an indicator of the response to treatment. The number of previous treatment lines in RRMM is an indicator of treatment resistance. [21]. In Rodon et al.’s study, the ORR was highest, but the patients included in this study had fewer prior treatment lines. The ORR decreases as the number of treatment lines increases.

The high efficacy of bendamustine in patients with extramedullary involvement compared to those without extramedullary involvement was effective for including it in the triple combination in our study. According to the results we obtained from these studies, we thought that the BPD treatment could be beneficial. Considering that our patients consisted of patients who received a median of four lines of treatment before BPD, BPD seems to have improved the prognosis. When our results are compared with clinical trial and real-life data studies, it will be understood that BPD treatment is an effective and safe treatment option. Our study adds to this evidence base, suggesting that our experimental treatment may be an effective alternative for RREMM patients who have failed previous lines of therapy. Our study, in comparison, focuses on a novel treatment for RREMM, providing additional therapeutic options for this difficult-to-treat population.

We examined the outcomes of patients with RREMM who underwent BPD treatment. The study yielded an overall response rate (ORR) of 54%, a two-year survival rate of 82%, and a two-year progression-free survival rate of 71.3%. These findings indicate that BPD treatment may offer a promising therapeutic approach for patients with RREMM.

The number of patients is clearly a limitation of our study. The major limitation of small studies is that they can produce false-positive results, or they over-estimate the magnitude of an association.

## 6. Conclusions

BPD regimens may present a feasible therapeutic approach for patients with RREMM, demonstrating improved outcomes in comparison to some other frequently utilized treatments within this patient population. To corroborate these findings and explore additional treatment alternatives for this patient group, larger prospective studies are required.

## Figures and Tables

**Table 2:** Sociodemographic and clinical characteristics of the patients.

	*n*	%
Gender		
Male	5	45.5
Female	6	54.5
ECOG * score		
0	1	9.1
1	6	54.5
2	4	36.4
Cytogenetic features at the time of diagnosis		
Normal	8	72.7
Del 17p	2	18.2
t(4;14)	1	9.1
Bortezomib use before BPD treatment	11	100
Lenalidomid use before BPD treatment	11	100
ASCT before BPD treatment		
No	3	27.3
Yes	8	72.7
Treatment response status after 8th cycle BPD		
VGPR	1	9.1
PR	5	45.4
SD	2	18.2
PD	3	27.3
Mortality		
Alive	9	81.8
Exitus	2	18.2
	Mean ± SD	Median (min–max)
Age	62.36 ± 7.50	66.00 (48–70)
Follow-up time (month)	19.8 ± 14.4	15 (4–36)
Median survival times (month)	25 ± 17.6	23 (4–43)
Number of previous treatment lines (including ASCT)	3.81 ± 0.75	4.00 (3–5)

VGPR very good partial response, PR partial response, SD stable disease, and PD progressive disease. * Eastern Cooperative Oncology Group.

**Table 3:** Various combination treatments used for RRMM.

Study Characteristics	Age	Patient Characteristics (RRMM)	Combination	Results of Study
Kumar et al., 2022 [21], phase II trial, *n* = 28	Median age 54 years (range 30–76 years)	Received at least two lines of therapy, refractory to both lenalidomide and bortezomib. Patients had a median of 3 prior treatment lines (range, 2 to 6 lines)	Bendamustine 120 mg/m^2^ day 1, pomalidomide 3 mg days 1–21, and dexamethasone 40 mg days 1, 8, 11, 22	Median follow-up of 8.6 months, ORR 57.6%, BPD showed better results in EMM patients
Musto et al., 2015 [16], retrospective, real-life analysis, *n* = 78	Median age 65 years (range 38–84 years)	Received at least one lines of therapy and median number of prior lines of therapy was 4	Bendamustine + bortezomib(*n* = 18), bendamustine + lenalidomid (*n* = 16), bendamustine + steroids (*n* = 39)	Median follow-up of 8 months, ORR were 39% in the bendamustine + bortezomib arm, 62% in the bendamustine + lenalidomide arm, and 10% in the bendamustine + steroid arm
Richardson et al., 2014 [17], phase II trial, *n* = 221	Median age 63 years (range 34–88 years)	Received ≥2 prior therapies (including lenalidomide and bortezomib) and median number of prior lines of therapy was 5	Pomalidomide 4 mg days 1–21 with low-dose dexamethasone (40 mg/week) (*n* = 113) or alone pomalidomide 4 mg days 1–21 (PD/P)	Median follow-up of 9.4 months, ORR were 33% in the pomalidomide, low-dose dexamethasone arm and 18% in the pomalidomide arm (*p* = 0.013)
Rodon et al., 2015 [18], phase II study, *n* = 73	Median age 76 years (range 66–86 years)	All patients had received only one line of therapy	Bendamustine 70 mg/m^2^ on days 1 and 8, IV bortezomib 1.3 mg/m^2^ on days 1, 8, 15 and 22, and oral dexamethasone 20 mg on days 1, 8, 15 and 22 (BVD)	Median follow up of 15.7 months, ORR were 69.8%, the median time from start of first-line therapy to the initial dose of chemotherapy of the BVD regimen was 29 months
Ludwig et al., 2014 [19], phase II study, *n* = 79	Median age 64 years	Patients had a median of 2 prior treatment lines (range, 1 to 6 lines)	Bendamustine 70 mg/m^2^ days 1 and 4; bortezomib 1.3 mg/m^2^ intravenously days 1, 4, 8, and 11; and dexamethasone 20 mg days 1, 4, 8, and 11 once every 28 days (BVD)	ORR was 60.8%, median time of follow-up was 13.7 months
Lentzsch et al., 2012 [20], phase 1/2, *n* = 29	Median age 63 years	Patients had a median of 3 prior treatment lines (range, 1 to 6 lines)	Bendamustine 75 mg/m^2^ (days 1 and 2), lenalidomide 10 mg (days 1–21), and dexamethasone 40 mg (weekly) of a 28-day cycle(BLD)	ORR was 52%, median time of follow-up was 13 months
